# Waist-To-Height Ratio Is a More Accurate Tool for Predicting Hypertension Than Waist-To-Hip Circumference and BMI in Patients With Type 2 Diabetes: A Prospective Study

**DOI:** 10.3389/fpubh.2021.726288

**Published:** 2021-10-07

**Authors:** Fatemeh Moosaie, Seyede Marzie Fatemi Abhari, Niloofar Deravi, Arman Karimi Behnagh, Sadaf Esteghamati, Fatemeh Dehghani Firouzabadi, Soghra Rabizadeh, Manouchehr Nakhjavani, Alireza Esteghamati

**Affiliations:** ^1^Endocrinology and Metabolism Research Center (EMRC), School of Medicine, Vali-Asr Hospital, Tehran University of Medical Sciences, Tehran, Iran; ^2^Department of Pediatrics, Imam Ali Hospital, Alborz University of Medical Sciences, Karaj, Iran; ^3^Student Research Committee, School of Medicine, Shahid Beheshti University of Medical Sciences, Tehran, Iran; ^4^Endocrine Research Center, Institute of Endocrinology and Metabolism, Iran University of Medical Sciences, Tehran, Iran

**Keywords:** hypertension, type 2 diabetes, body mass index, waist-to-height ratio, waist-to-hip ratio

## Abstract

**Background:** Anthropometric measures [i.e., body mass index (BMI), waist-to-hip ratio (WHR), and waist-to-height ratio (WHtR)] have been used as prediction factors for incident hypertension. However, whether any of these measures is superior to another in the matter of accuracy in predicting hypertension in diabetic patients has been controversial. The present prospective study aimed to determine whether WHtR is a more accurate tool for predicting hypertension than WHR and BMI in patients with type 2 diabetes.

**Methods:** The study population consisted of 1,685 normotensive patients with type 2 diabetes. BMI, WHR, and WHtR were assessed at baseline and followed up for hypertension incidence for a mean of 4.8 years. A cox regression analysis was performed to assess the association between anthropometric measures (i.e., BMI, WHR, and WHtR) and incident hypertension during the follow-up period. The area under the ROC curve analysis was performed and optimal cutoff values were calculated for each anthropometric measure for hypertension prediction.

**Results:** WHtR and BMI were significantly associated with an increased incidence of hypertension (HR = 3.296 (0.936–12.857), *P* < 0.001, and HR = 1.050 (1.030–1.070), *P* < 0.001, respectively). The discriminative powers for each anthropometric index for hypertension were 0.571 (0.540–0.602) for BMI, 0.518 (0.486–0.550) for WHR, and 0.609 (0.578–0.639) for WHtR. The optimal cutoff points for predicting hypertension in patients with type 2 diabetes were 26.94 (sensitivity = 0.739, specificity = 0.380) for BMI, 0.90 (sensitivity = 0.718, specificity = 0.279) for WHR, and 0.59 (sensitivity = 0.676, specificity = 0.517) for WHtR.

**Conclusion:** WHtR was a more accurate tool for predicting hypertension compared to WHR and BMI in patients with type 2 diabetes.

## Introduction

Diabetes mellitus is among the most common diseases worldwide that significantly affects the cardiovascular system. The main cause of death among patients with diabetes is cardiovascular diseases (CVD) (1–3). Diabetes mellitus is associated with several major cardiovascular complications, including coronary artery disease (myocardial infarction and angina pectoris), heart failure, stroke, and peripheral artery disease. Besides, cardiovascular risk factors increase the risk of developing these complications. Hypertension (HTN), a highly prevalent cardiovascular risk factor in patients with diabetes (present in 35% of men and 46% of women with diabetes), causes three-quarters of the cardiovascular deaths in these patients (4). The hypertension diagnosis is confirmed in a gradual process, requiring multiple careful blood pressure assessments at different times (5). Late diagnosis of hypertension extends the harmful effects of high blood pressure on the cardiovascular system, promoting the onset of irrecoverable sequelae (6).

Since obesity, particularly abdominal obesity, plays a significant role in the etiology of hypertension, using anthropometric indicators of adiposity may help screen patients who are at a higher risk for developing hypertension. The identified individuals could be referred to health centers with better hypertension monitoring systems earlier, and therefore, the early diagnosis of hypertension optimizes the opportunity for secondary prevention measures (7).

Body mass index (BMI), the standard general obesity measure, is the most commonly used indicator in hypertension prediction and screening. The adipose tissue distribution is a significant factor in developing cardiovascular diseases compared to total body fat. Thus, several measures that consider the distribution of adipose tissue, including WHR (waist-to-hip ratio) and WHtR (waist-to-height ratio) have been developed (8). Researchers have conducted numerous studies to assess the associations between the various obesity indicators and hypertension in recent decades. Cuban and Japanese studies have suggested BMI as the best single predictor of hypertension (9, 10). In contrast, an Iranian cross-sectional study suggested WHR combined with BMI as the best predictor of hypertension in women (11). A 2006 meta-analysis reported the superiority of centralized obesity measures, especially WHtR over BMI for CVD risk detection (12). However, another meta-analysis in 2008 reported that none of the anthropometric variables were systematically better than the others at hypertension discrimination (13). Therefore, there is no consensus in the literature over the most accurate measure for hypertension prediction. The present prospective study aimed to determine whether WHtR is a more accurate tool for predicting HTN than WHR and BMI in patients with type 2 diabetes.

## Materials and Methods

### Participants

**Figure 1:**
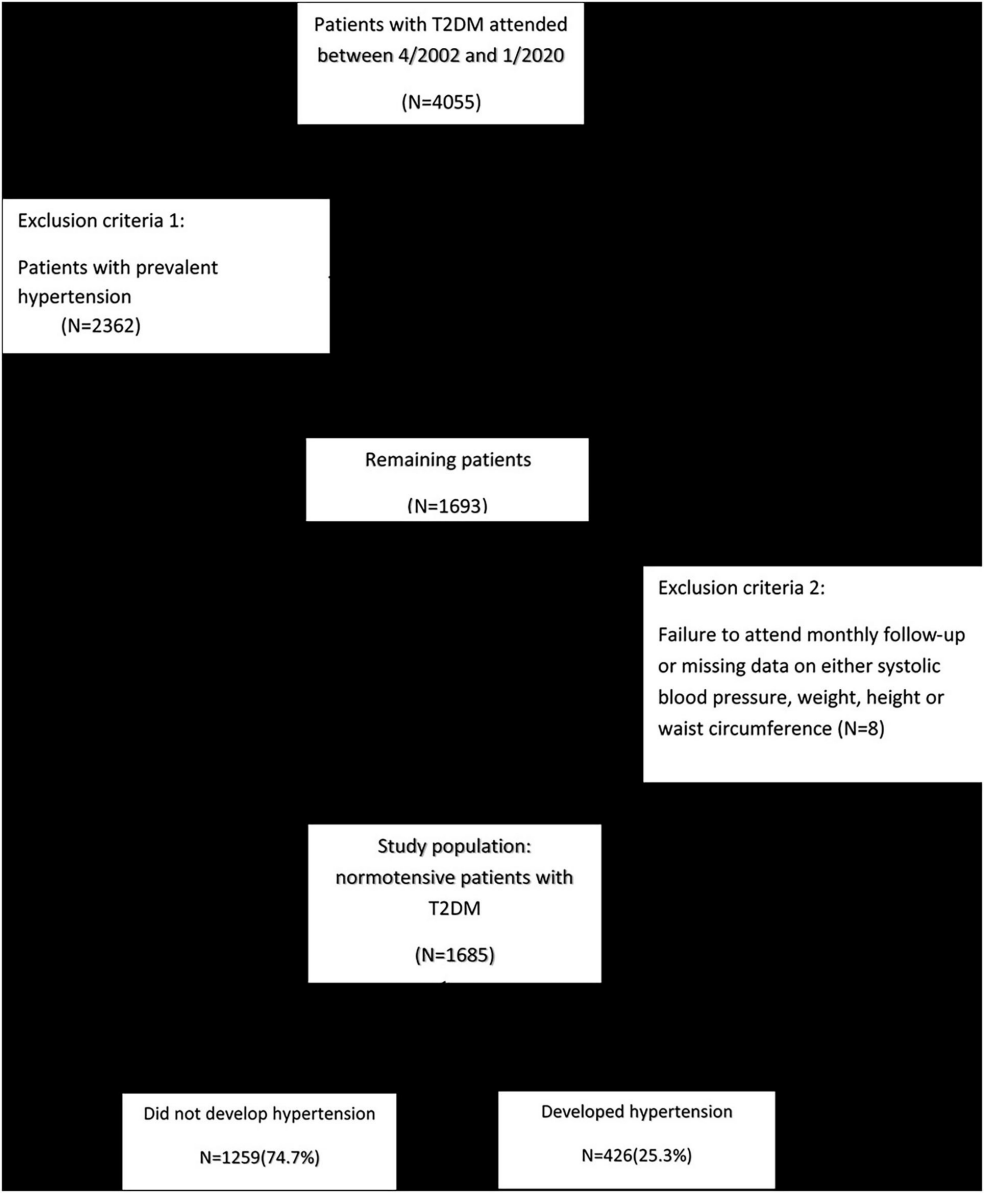
Study population selection flow chart; T2DM = type 2 diabetes mellitus.

Four thousand fifty-five patients who were previously diagnosed with type 2 diabetes participated in this study from April 2002 to January 2020. All patients attended the endocrinology clinic of Vali-Asr Hospital, a tertiary center affiliated with Tehran University of Medical Sciences. After excluding the patients with previously diagnosed hypertension (*N* = 2,362), the ones who failed to attend monthly follow-up, or had missing data on systolic blood pressure, weight, height, or waist circumference (*N* = 8), the final study population consisted of 1,685 normotensive patients with T2DM (Figure 1). The patients' anthropometric measures, including BMI, WHR, and WHtR, were assessed at baseline. Moreover, each participant was followed-up for hypertension for a mean follow-up period of 4.8 years at some point between April 2002 to January 2020. This study was in full compliance with the Declaration of Helsinki and was reviewed and approved by the Ethics Committee of Tehran University of Medical Sciences.

### Data Collection

At baseline, all participants were interviewed by a trained interviewer. Data collection at baseline consisted of the following parameters: identification and sociodemographic characteristics, including age, gender, education, marital status, lifestyle habits such as smoking, self-reported health conditions, and specifically, the use of antihypertensive drugs, past medical history, including any physical or mental conditions, and objective measures including, blood pressure and body weight. Data collection during the follow-up period was limited to the following parameters: identification (only age and gender), self-reported health conditions (use of antihypertensive medications), and objective measures (only blood pressure). In this study, current smokers were defined as individuals who had smoked a minimum of 100 cigarettes in their lifetime and continued smoking during the follow-up period. A team of trained and closely observed researchers using standard equipment was responsible for data collection at all times.

### Anthropometric Measurements

Weight was measured using a portable digital scale with a precision of 0.1 kg in minimally clothed participants without shoes. Height was measured using a standard protocol with a tape measure. The body mass index (BMI) was calculated as weight (kilograms) divided by squared height (m^2^). The BMI values were categorized according to the World Health Organization (WHO)'s classification: <18.5 kg/m^2^ (underweight), 18.5 to 24.9 kg/m^2^ (normal weight), 25 to 29.9 kg/m^2^ (overweight) and ≥30 kg/m^2^ (obese). Waist circumference (WC) was measured at the end of a normal expiration. The tape was positioned horizontally at the midway between the iliac crest and the inferior border of the last rib according to standard WHO protocol (14). Likewise, Hip circumference was measured at the widest circumference of buttock while the tape was horizontal and parallel to the ground. During these procedures, the subjects were standing and wearing minimal clothing. All the measurements were performed with a precision of 0.1 cm. In order to minimize observer error, all measurements were carried out by the same technician. The waist-to-height (WHtR) and waist-to-hip ratio (WHR) were calculated by dividing waist (cm) by height (cm) and hip circumference (cm), respectively.

### Laboratory Measurements

From each subject, 10 mL of venous blood was drawn after 12–14 h of overnight fasting. The samples were kept in cold biochemistry tubes with a temperature of 4–8°C and sent to the representative calibrating laboratories within 4 h, where the samples were immediately centrifuged (1,500 RPM, for 10 min at standard room temperature 21°C). Then, the extracted serum was used for laboratory evaluations. Enzymatic calorimetric methods using the glucose oxidase test were implemented to measure the fasting plasma glucose (FPG) and 2 h post-prandial glucose (2 hPPG). Glycated hemoglobin (HbA1c) was measured using high-performance liquid chromatography. By using enzymatic methods, the serum concentrations of triglycerides, low-density lipoprotein cholesterol (LDL-C), high-density lipoprotein cholesterol (HDL-C), and total cholesterol were determined. The commercially-available kits, distributed by the central reference laboratory (Tehran, Iran), have passed all the quality control procedures.

### Definition of Hypertension

Blood pressure (BP) was measured on the left arm by using a mercury sphygmomanometer (Reishter, Germany) with an appropriate cuff size according to the subjects' arm circumference. The procedure was performed in the sitting position after a 10-min resting period, and all the subjects were asked not to smoke or consume caffeine 30 min preceding the procedure commencement. BP was measured twice, with a 10-min interval between the two measurements. During this interval, the patients were asked to remain seated with their left hand placed at the heart level and their palm in the upward-facing position. The mean of the two measured values were considered as the patient's BP. Auscultated systolic blood pressure (SBP) was defined as the first Korotkoff sound heard during the cuff deflation, while diastolic blood pressure (DBP) was marked by the disappearance of the last Korotkoff sound. Patients using antihypertensive drugs or subjects with an SBP higher than 140 mmHg or a DBP higher than 90 mmHg were classified as hypertensive (15).

### Statistical Analysis

SPSS version 24.0 (SPSS Inc., Chicago, IL, USA) was employed for statistical analysis. We used Kolmogorov–Smirnov and Shapiro-Wilk normality tests, P-P plot, and histogram to test the normality of our study population. The null hypothesis was rejected for all the variables; thus, they were normal. To assess the association between different variables and incident hypertension, uni-variable analysis of potential continuous and categorical risk factors was performed using *t*-test and chi-square, respectively. The data were reported as mean ± standard deviation (SD) for continuous variables and as proportions for categorical variables. Cox regression analysis was performed to assess the association between anthropometric measures (i.e., BMI, WHR, and WHtR) and incident hypertension during the follow-up period. Hazard ratios (HR) were adjusted for the confounding variables (i.e., age, gender, history of coronary artery disease (CAD), SBP, DBP, FPG, and family history of hypertension). The Cox regression analysis was also used to calculate HRs of hypertension and their 95% CIs with reference to a normal BMI and the first quartile of WHR and WHtR. The adjustment was performed for age, gender, history of CAD, SBP, DBP, FPG, history of CAD, and family history of hypertension. The discriminative powers of BMI, WHR, and WHtR were assessed by the area under the ROC curve analysis in the group with incident hypertension. The area under the ROC curve of the baseline BMI, WHR, WHtR, and their 95% confidence intervals (CIs) were reported. Optimal cutoff values were calculated for each aforementioned anthropometric measure for the diagnosis of hypertension. Youden index was employed for calculating the optimal cutoff value. The sensitivity and specificity of each of the anthropometric measures were determined based on the calculated cutoff values. The risk of incident hypertension was compared among the BMI categories (i.e., underweight, normal, overweight, and obese) and the quartiles of WHR and WHtR. A two-sided *p*-value < 0.05 was considered necessary to reject the null hypothesis.

## Results

### Baseline Characteristics

A total of 1,685 participants were enrolled in this study. The mean follow-up period was 4.8 years, and throughout this period, 426 participants (25.3%) developed hypertension. Table 1 summarizes the baseline characteristics of all participants based on the development of HTN. A comparison of study subgroups at baseline revealed higher means for age, SBP, DBP, 2-hpp, CAD prevalence, and anthropometric measures, as well as a higher frequency of HTN in family members of the subjects who developed HTN. Furthermore, the two groups differed significantly in terms of BMI and WHtR. The frequency of obese subjects was significantly higher in HTN group (42.8 vs. 37%). Also, a higher proportion of patients who developed HTN were in the fourth quartiles of WHtR (37.6 vs. 24.9%) (Table 1).

**Table 1:** Baseline characteristics of the study population, based on hypertension status at the time of follow-up.

		**Did not develop hypertension (*N* = 1259)**	**Developed hypertension(*N* = 426)**	***P*-value**
Age(yr)		54.82 (± 11.12)	58.95 (± 9.15)	<0.001
Gender (M)		614 (48.8%)	152 (35.7%)	<0.001
Waist/height ratio		0.60 (± 0.07)	0.63 (± 0.07)	<0.001
Waist/hip ratio		0.93 (± 0.06)	0.94 (± 0.06)	0.130
Weight (kg)		77.66 (± 14.85)	77.92 (± 14.86)	0.756
BMI (kg/m^2^)		29.04 (± 4.96)	30.24 (± 5.23)	<0.001
SBP (mmHg)		124.92 (± 13.53)	130.01 (± 9.65)	0.041
DBP (mmHg)		77.99 (± 6.31)	79.18 (± 9.00)	0.038
TC (mg/dL)		183.03 (± 41.96)	181.14 (± 47.97)	0.468
TG (mg/dL)		173.03 (± 109.95)	182.64 (± 98.95)	0.110
HDL (mg/dL)		45.30 (± 12.00)	45.46 (± 13.79)	0.823
LDL (mg/dL)		104.14 (± 33.42)	101.38 (± 37.07)	0.152
FPG (mg/dL)		156.54 (± 52.51)	163.59 (± 52.70)	0.170
2hpp (mg/dL)		215.77 (± 84.37)	236.85 (± 86.05)	<0.001
HbA1c (mg/dL)		7.74 (± 4.97)	7.78 (± 1.50)	0.874
Current smokers, *n* (%)^*^		28 (2.2)	36 (12.1)	0.033
Family history of HTN, yes, *n* (%)		434 (34.7)	240 (56.7)	<0.001
History of CAD, yes		234 (18.6)	120 (28.2)	<0.001
BMI categorized*n* (%)	Underweight	5 (0.4)	1 (0.2)	<0.001
	Normal	239 (19.0)	49 (11.5)	
	Overweight	558 (44.3)	186 (43.7)	
	Obese	457 (36.3)	190 (44.6)	
Waist-to-hip ratio quartiles*n* (%)	Q1	93 (7.4)	27 (6.3)	0.873
	Q2	296 (23.5)	105 (24.6)	
	Q3	408 (32.4)	136 (31.9)	
	Q4	462 (36.7)	157 (37.1)	
Waist-to-height ratio quartiles*n* (%)	Q1	188 (14.9)	33 (7.7)	<0.001
	Q2	392 (31.1)	94 (22.1)	
	Q3	366 (29.1)	139 (32.6)	
	Q4	313 (24.9)	160 (37.6)	

* *n(%): Number of patients who matched each category and the percentage in each patient group. BMI, Body mass index; SBP, Systolic blood pressure; DBP, Diastolic blood pressure; TC, Total cholesterol; TG, Triglycerides; HDL, High density lipoprotein; LDL, Low density lipoprotein; FPG, Fasting plasma glucose; 2 hpp, 2 h post prandial; HbA1c, Hemoglobin A1c; HTN, Hypertension; CAD, Coronary artery disease*.

### Development of Hypertension in Patients With T2DM According to WHtR, WHR, and BMI

After adjusting for age, gender, history of CAD, SBP, DBP, FPG, and family history of HTN, WHtR and BMI were found to be significantly associated with an increased risk of hypertension (HR = 3.296, 95% CI: 0.936–12.857, *P* < 0.001, and HR = 1.050, 95%CI: 1.030–1.070, *P* < 0.001, respectively). However, this association was not significant for WHR (Table 2). In another adjusted cox regression model assessing the association between incident hypertension and different categories of BMI (i.e., underweight, normal, overweight, and obese), and four quartiles of WHR and WHtR, a higher WHtR at the baseline, was positively and significantly associated with the development of hypertension in a ratio-dependent manner (highest vs. lowest quartile HR 1.936, 95%CI: 1.306–2.871) (Table 3).

**Table 2:** Cox regression analysis determining the association between BMI, waist-to-hip ratio, and waist-to-height ratio with incident hypertension during the follow-up period; results are adjusted for age, gender, history of CAD, SBP, DBP, FPG and family history of HTN.

	**HR (95% CI)**	***P*-value**
BMI	1.050 (1.030–1.070)	<0.001
Waist-to-hip ratio	2.604 (0.605–10.477)	0.159
Waist-to-height ratio	3.296 (0.936–12.857)	<0.001

*BMI, Body mass index*.

**Table 3:** Hazard ratio of incident hypertension based on waist-to-height ratio, waist-to-hip ratio and BMI during the follow-up period; Data are presented as HR (95% CI); Results are adjusted for age, gender, history of CAD, SBP, DBP, FPG and family history of HTN.

	**Quartile 1**	**Quartile 2**	**Quartile 3**	**Quartile 4**	***P*-value**
**Waist-to-hip ratio**	Ref	1.247 (0.812–1.914)	1.039 (0.680–1.587)	1.246 (0.810–1.917)	0.328
**Waist- to-height ratio**	Ref	1.354 (0.905–2.025)	1.534 (1.034–2.276)	1.936 (1.306–2.871)	0.003
	**Underweight**	**Normal**	**Overweight**	**Obese**	**P-value**
**BMI**	0.664 (0.091–4.867)	Ref	1.394 (1.013–1.919)	1.793 (1.294–2.486)	0.002

*BMI, Body mass index*.

### ROC Analysis of WHtR, WHR, and BMI for Prediction of Hypertension in Patients With T2DM

**Figure 2:**
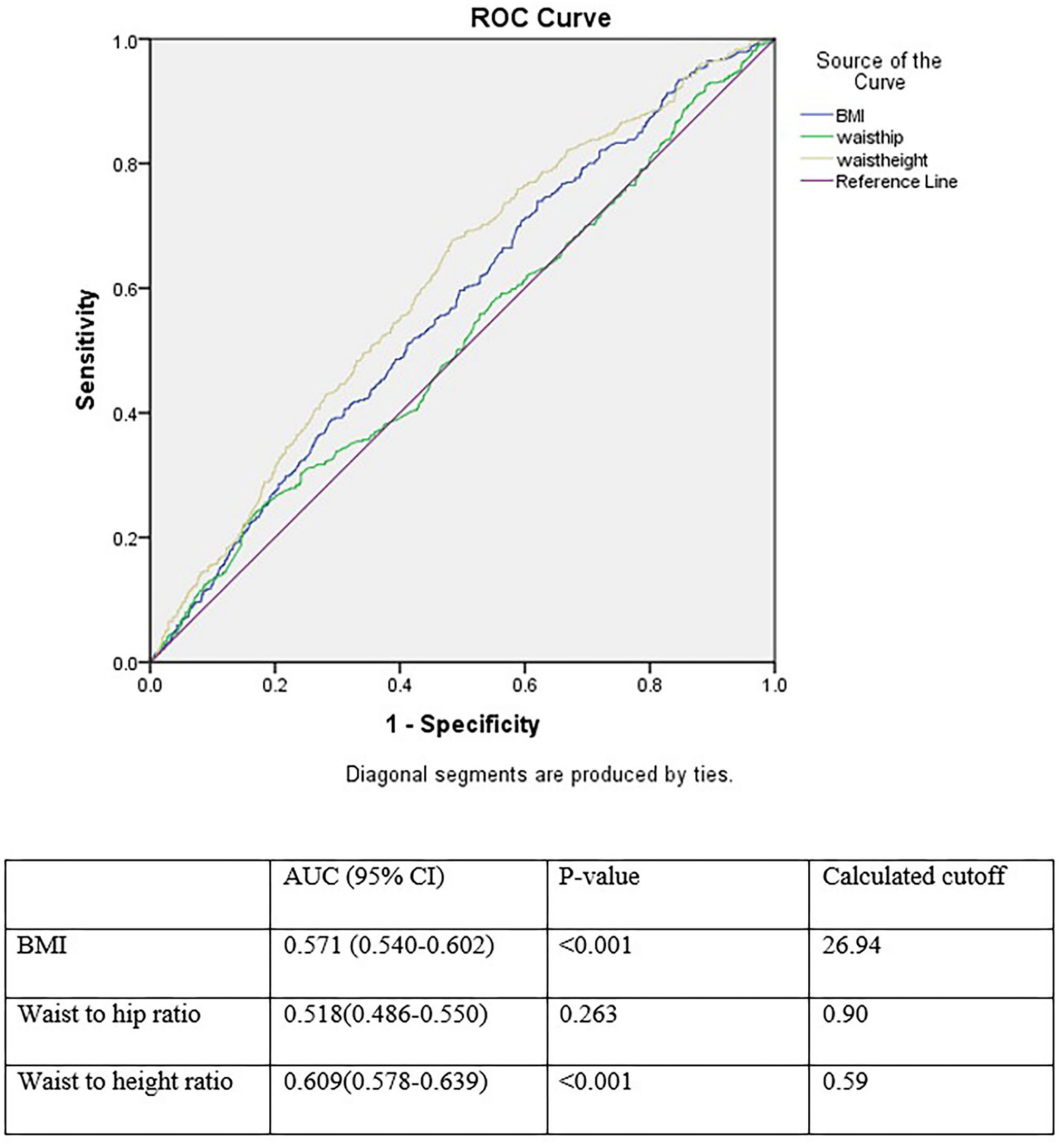
Receiver operating characteristics curve analysis for comparison of BMI, waist to hip ratio, and waist to height ratio as an index for incident hypertension.

Figure 2 shows the areas under the ROC curve (AUC) of the anthropometric measurements for the prediction of HTN. All anthropometric indices, especially WHtR, exhibited a predictive power in detecting the incidence of HTN in patients with T2DM. The discriminative powers of each anthropometric index for HTN were 0.571 (95% CI: 0.540–0.602) for BMI, 0.518 (95% CI: 0.486–0.550) for WHR, and 0.609 (95% CI: 0.578–0.639) for WHtR. The optimal cutoff points for predicting HTN in patients with T2DM were 26.94 (sensitivity = 0.739, specificity = 0.380) for BMI, 0.90 (sensitivity = 0.718, specificity = 0.279) for WHR, and 0.59 (sensitivity = 0.676, specificity = 0.517) for WHtR.

## Discussion

More than 4.5 million Iranian adults were diagnosed with diabetes in 2011; this number is projected to rise to 9.2 million by 2030. This increase emphasizes the significance of type 2 diabetes, most importantly, when considering the burden of diabetes-related complications (16). Several studies have reported high prevalence of hypertension in patients with diabetes (17–22). The existence of hypertension in patients with diabetes significantly increases the risk of stroke, coronary artery disease, retinopathy, and nephropathy (23). Therefore, identifying the risk factors and predictors of hypertension is of high importance to diagnose, prevent, control, and treat this condition as soon as possible. Our results indicated higher means for age, TG, CAD prevalence, smoking status, and anthropometric measures, as well as a higher frequency of HTN in family members of the subjects who developed HTN. Additionally, the frequency of obese subjects was significantly higher in the HTN group. These results are in line with the data from previous studies on the associated risk factors of hypertension (24).

Furthermore, multivariate analysis in this study revealed that WHtR, unlike BMI and WHR, was significantly associated with increased risk of developing hypertension, subsequent to adjustment for age, gender, history of CAD, SBP, FBS, and TG. Moreover, the ROC analysis of anthropometric indices for prediction of hypertension demonstrated that all indices, especially WHtR, exhibited a statistically significant predictive power in detecting the incidence of HTN in patients with T2DM. Therefore, according to our data, WHtR is a better screening tool for HTN than WHR and BMI in Iranian men with type 2 diabetes. This finding is similar to the results of various studies on different ethnicities worldwide (7, 25–30). Three meta-analyses also supported WHtR as the most accurate anthropometric index for predicting hypertension (12, 31, 32).

WHtR can be considered as the best anthropometric index for screening for health risks. Height can predict hypertension and diabetes (33, 34), and the percentage of body fat is an independent risk factor for CVD (35). People with shorter stature have noticeably higher amounts of body fat than taller individuals with the same BMI (36). Individuals with similar WCs might not have a similar percentage of body fat if they are unequal in height. Therefore, WHtR can be a helpful predictor of hypertension by considering the impact of waist circumference and height on body fat composition (29).

In the current study, optimal cutoff for WHtR was calculated as 0.59. A recent Iranian study on anthropometric indices for obesity reported cutoff of 0.49–0.51 among adults (37). A cutoff of 0.5 for WHtR was first mentioned in a study by McCarthy HD et al. in 2006 on UK adolescents and children over two decades supporting the simple message: “Keep your waist circumference to less than half your height” (38). Hsieh et al. recommended a WHtR cutoff of 0.5 for Japanese men and women (39). Tseng et al. also reported a cutoff of 0.48–0.52 in both sexes and suggested that this is the clinical importance of WHtR as the same cutoff could be applied to different sexes and ethnicities (29). WHtR is the least expensive and a simple measure that has a strong relationship with cardiovascular diseases, with a similar optimal cutoff at 0.5 in both sexes and various ethnic groups. Due to these features, WHtR can be of great value to public health and can be utilized as a standard screening tool for more accurate epidemiological data comparisons between studies. The cutoff value of 0.5 can be simply memorized and instantly employed to explain the risk of cardio-metabolic diseases to the patients to determine if the WC is less than half of the height. Thus, no more complicated calculations are needed (as required for BMI) (29).

On the other hand, Cuban and Japanese studies suggested BMI as the single best indicator for developing hypertension (9, 10). Li et al. reported BMI and WC can predict incident hypertension more accurately than WHR, skinfold thickness, and WHtR in the Chinese population (8). A study on the Iranian population also reported that BMI is the best predictor of hypertension in men; BMI combined with WHR were the best predictors of hypertension in women (11). Another Iranian cross-sectional study also suggested WHR combined with BMI as the best predictor of hypertension in women (11). Rezende et al. suggested that both central obesity (WC and WHtR) and overall obesity (BMI) anthropometric indicators could be used in the Brazilian population to evaluate the risk of incident hypertension (40). Another Brazilian study, as well as a 2008 meta-analysis reported that all indices had similar performances in hypertension detection (13, 41). These differences might be due to the populations' varying characteristics, differences in sampling strategies, data collection quality, and the differences in operational definitions for abdominal and general obesity (42).

A screening measure should be both efficient and practical. BMI is calculated using weight and height. This measure does not reflect the distribution of individual's fat (general vs. abdominal obesity). Studies have shown that patients with the same BMI may have different waist circumferences. Since, people with abdominal obesity are more prone to cardiovascular diseases and hypertension (43), it is crucial to employ a measure that reflects WC and either WHR or WHtR. When comparing WHR with WHtR; WHtR seems to have several advantages over WHR. Firstly, as mentioned earlier people with shorter stature have noticeably higher amounts of body fat than taller individuals with the same BMI (36). Secondly, studies have reported that self-assessment of height is more accurate than weight (43). Thirdly, height has usually been shown to have inverse associations with cardiometabolic morbidity and mortality and this is probably because height, as well as having a major genetic component, can also reflect general early life exposures and WHR does not reflect body height (32, 44, 45). Furthermore, in women with T2DM, WHtR has been shown to be independently and better associated with elevated urinary albumin excretion rate, a common cardiovascular risk factor in diabetes (46), than WC, WHR, and BMI (47). It has also been reported to have the superiority of independent association with coronary artery disease and the highest magnitude of association than WHR, and BMI in patients with T2DM (48).

Hsieh et al. argued that among the Japanese men in the third quartile of WC (84.5 - 89 cm), shorter individuals were more prone to hypertension than the taller individuals (49). Furthermore, it is harder to measure hip circumference than WC, since there is a need to accurately identify the point of maximal protrusion of the buttocks in obese people (14). A former study argued that the area under the ROC for WHR for hypertension was reported as the lowest among anthropometric indices (50). This finding is in line with the results of our study. Most importantly, WHR and WC are not suitable measures due to the variously reported cutoffs for the two sexes (45, 51).

The current study had several strengths. Firstly, to the best of our knowledge, this is the first prospective cohort study comparing obesity anthropometric indices in patients with diabetes. Secondly, it used a prospective cohort design as well as a large population-based sample. Thus, there is a clear causality between incident hypertension and obesity anthropometric indices. Thirdly, professional interviewers performed several interviews. Anthropometric data were recorded through repeated measurements in accordance with a standard protocol. Such procedures helped to reduce bias. This study had several limitations. Firstly, since our study population was limited to the Iranian ethnicity, caution should be taken when extrapolating the results to other ethnicities. Secondly, since the participant's diet details were not available, we couldn't adjust for hypertension-related covariates, such as salt and fat intake. Thirdly, repeated measures of the anthropometric indices and other variables measured at baseline, were not available for all the patients, so we could not analyze the role of time-varying indices and adjust for the time-varying confounders.

## Conclusion

The present prospective study showed that WHtR is a more accurate screening tool for HTN than WHR and BMI in patients with type 2 diabetes. Our data reinforce the significance of including the most accurate anthropometric indices in the public health strategies to prevent and control the obesity epidemic and address the risk of developing hypertension. Therefore, WHtR could be recommended as a useful and accurate screening tool to predict hypertension due to its high discriminative power.

## Data Availability Statement

The original contributions presented in the study are included in the article/supplementary material, further inquiries can be directed to the corresponding authors.

## Ethics Statement

The studies involving human participants were reviewed and approved by the Ethics Committee of Tehran University of Medical Sciences. The patients/participants provided their written informed consent to participate in this study.

## Consent for Participate

All participants voluntarily agreed to participate in this research study.

## Author Contributions

All authors listed have made a substantial, direct and intellectual contribution to the work, and approved it for publication.

## Conflict of Interest

The authors declare that the research was conducted in the absence of any commercial or financial relationships that could be construed as a potential conflict of interest.

## Publisher's Note

All claims expressed in this article are solely those of the authors and do not necessarily represent those of their affiliated organizations, or those of the publisher, the editors and the reviewers. Any product that may be evaluated in this article, or claim that may be made by its manufacturer, is not guaranteed or endorsed by the publisher.

## Abbreviations

HTN: Hypertension
T2DM: Type 2 Diabetes
BMI: Body mass index
WHtR: Waist-to-height ratio
WHR: Waist-to-hip ratio
SBP: Systolic blood pressure
DBP: Diastolic blood pressure
TC: Total cholesterol
TG: Triglycerides
HDL: High density lipoprotein
LDL: Low density lipoprotein
FPG: Fasting plasma glucose
2hpp: 2-hour post prandial
HbA1c: Hemoglobin A1c
HTN: Hypertension
CAD: Coronary artery disease.
